# Nepal's experience in implementing the federal government system: Assessment of law-making by the local governments of Kaski district, Nepal

**DOI:** 10.1016/j.heliyon.2024.e26250

**Published:** 2024-02-10

**Authors:** Girdhari Dahal

**Affiliations:** Department of Political Sciences, Prithvi Narayan Campus, Tribhuvan University, Pokhara, Nepal

**Keywords:** Constitution, Federalism, Law-making, Local government, Power distribution

## Abstract

The Constitution of the Federal Republic of Nepal 2015 institutionalizes the sovereign right of the people and the right to autonomy, shared rule and self-rule via the three levels of government: federal, provincial, and local. Accordingly, to deliver service to the local residents and to assure the residents of the practice of self-rule, the constitution has given state powers to the local levels with 22 exclusive mandates and 15 concurrent competencies and mandates. However, being the new practice in the Nepalese context, for implementing the delegated authority, related laws are to be formulated by the concerned local level. In this study, I assessed if the five local levels of Kaski district: Pokhara Metropolitan, Annapurna Rural Municipality, Machhapuchhre Rural Municipality, Madi Rural Municipality, and Rupa Rural Municipality in the first five years of their establishment formulated the requisite laws concerning the 22 local-level powers. I conducted a questionnaire survey and in-person interviews with the elected members of the local levels. The result revealed that during the first five years of operation (2017–2022), Pokhara Metropolitan formulated 98 laws, Annapurna Rural Municipality made 34 laws, Machhapuchhre Rural Municipality made 54 laws, Madi Rural Municipality made 88 laws, and Rupa Rural Municipality made 56 laws. The laws formed by the five local levels were of mainly two categories. The laws of the first category were related to the legal set-up of the local governments for the smooth functioning of administrative affairs which were almost identical across the five local levels. The laws of the second category were specific to the local level and thus apparently distinctive from each other, however, those laws were aimed towards providing service to the local residents, meeting the distinctive development aspirations of the local people and fulfilling the needs of local self-governance. Despite the deficiency of expertise in law-making and the limitation of resources, the local levels have laid a foundation for the functioning of local-level government. Although the local units of the Kaski district have put their best effort into formulating laws, they are yet insufficient to ensure the delivery of all the 22 constitution-delegated rights of the local residents. Therefore, the newly elected government should keep priority in formulating the remaining laws not only for the smooth functioning of the local government but also to assure the residents of the aspiration of self-governance and the constitution-granted rights.

## 1 Introduction

Among the different models of government, in the Federal system, there are multiple tiers of government such as federal or national government, regional, state or provincial government, local government, etc. with clearly defined state power. Indeed, the concept of the federal system is based on the principle of distributing or sharing power and authority between two or three spheres of government [[1], [2], [3],^20^,^33^]. Universally, there are two forms of federal government framework being adopted by different federal nations: three-tier government and two-tier government [4,5]. Among the 29 countries adopting federalism [6,7], federal countries like India, South Africa, Belgium, Australia, Switzerland, and Germany are found to have three tiers of government while federal countries like the USA, Pakistan, and UAE are found to have two tiers of government. In the two-tier federal system, the federal government is at the central level. It is usually responsible for a few subjects of common national interest. In contrast, the local government operates at the local (grassroots) levels that look after much of the day-to-day administering of their local unit [[4], [5], [6]]. In the three-tier federal system, in addition to the federal and local government, there is a provincial government that operates at the provincial level [[8], [9], [10], [11]].

The Constitution of Nepal 2015 restructures the country into a federal republic from a long-practised unitary form of government. Since then, following the suggestion of the high-level recommendation commission on state restructuring [12], which rigorously studied the federal practice of 28 federal nations, and from the political aspirations of major political parties in the constituent assembly, Nepal has decided to follow three tiers of government system, e.g. Central government as Federal, 7 Provincial governments, and 753 Local governments [13]. The main aim of restructuring the country into a federal system is to practice power distribution among the three levels of government with the expectation that the three-tier government system would be instrumental in resolving regional inequality/imbalance and reducing economic, social, and religious discrimination. The constitution of Nepal 2015 has guaranteed 31 fundamental rights to its citizens.

. Moreover, the Constitution clearly defines and distributes state powers among the three tiers of government [[14], [15], [16]]. The federal government has 35 rights, the provinces have 21 rights, and local governments have 22 rights. In addition, there are some concurrent rights among the federal, provincial, and local governments [16,17].

The constitution of Nepal 2015 considers that among the three tiers of government, the federal government is at the central level, the provincial government operates at the provincial level while the local level operates at the grassroots level working directly with the people to provide services and facilities to the local people. At the same time, this government is the regime of the people as people can access services and seek their accountability and transparency. Although the local government has been in practice in Nepal for long [18,19], the current form of local government has been in practice since the constitution of Nepal 2015 came into effect [20]. As defined in Schedule 8 of the constitution, the local government has the authority to exercise powers that are specific to the particular region which have direct and/or indirect links to the development, conservation, and livelihood of residents. This covers topics like providing public services and fostering the conservation of languages, cultures, and festivals, operation of local FM stations, conservation of local biodiversity [21] and so on [Table 1]. The local level is a people-orientated government and addresses people's desire to use their natural and human resources for their overall development through the provision of products and service delivery daily. Therefore, the current new form of local government is in charge of numerous local matters, such as the political role, administrative function, development activities, and service delivery at the local level. As a result, the local government is playing a crucial role in improving the residents' living conditions.

**Table 1:** List of Local-Level Powers granted by the constitution of Nepal 2015 to the local government.

S. N.	Matters
1	Town police
2	Cooperative institutions
3	Operation of F.M.
4	Local taxes [wealth tax, house rent tax, land and building registration fee, motor vehicle tax], service charges, fees, tourism fees, advertisement tax, business tax, land tax [land revenue], penalty, entertainment tax, land revenue collection
5	Management of the Local services
6	Collection of local statistics and records
7	Local-level development plans and projects
8	Basic and secondary education
9	Basic health and sanitation
10	Local market management, environment protection, and biodiversity
11	Local roads, rural roads, agro-roads, irrigation
12	Management of Village Assembly, Municipal Assembly, District Assembly, local courts, mediation, and arbitration [251]
13	Local records management
14	Distribution of house and land ownership certificates
15	Agriculture and animal husbandry, agro-products management, animal health, cooperatives
16	Management of senior citizens, persons with disabilities, and the incapacitated
17	Collection of statistics on the unemployed
18	Management, operation, and control of agricultural extension
19	Water supply, small hydropower projects, alternative energy
20	Disaster management
21	Protection of watersheds, wildlife, mines, and minerals
22	Protection and development of languages, cultures, and fine arts

Laws are a prerequisite for the smooth functioning of any system of governance in the federal system. However, Nepal is facing different challenges of governance in both law formulation and implementation. As Nepal has embraced a federal republic system, efforts and achievements that should have happened in law formulation, implementation, and enforcement have not been satisfactory. Devkota [22] argues that in recent years Nepal's law-making processes at all levels of government have been rife with issues. Additionally, parliaments across the three levels are not very dynamic, and even parliamentary committees are not functioning as expected. Bills have been stuck for years. Restoring local governments' ability to pay for necessary infrastructure, hiring personnel, recruitment, law enforcement, etc. are matters of concern. Therefore, to increase citizens' capacities and give them more influence over local government representatives for responsive governance, local governments need to enact laws to institutionalize the governance system, promote public participation in decision-making, particularly for empowering marginalized groups, and promote economic development [23,24]. This makes the local government the closest unit accessible and sensitive to the concerns of grassroots communities.

After the periodic local elections in 2017 and 2022, in five years, a formal law-making practice has been established in Nepal [[25], [26], [27], [28], [29]].

**Fig. 1:**
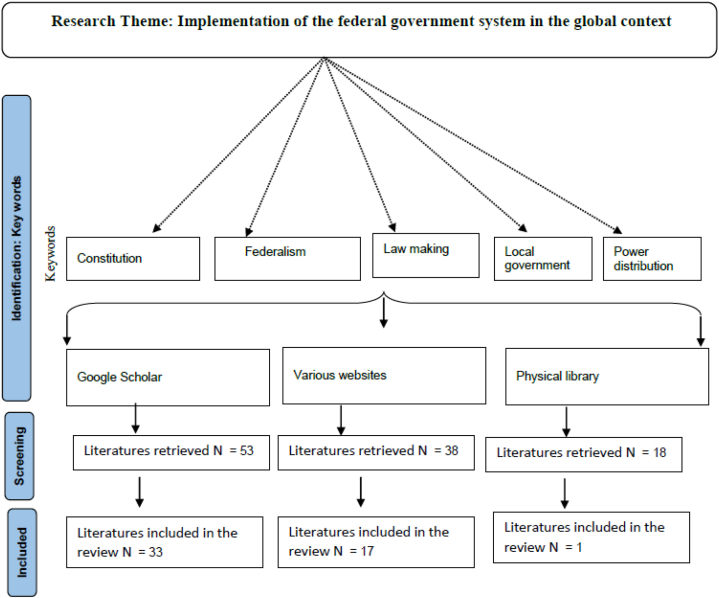
A flow chart showing the methodologies of the literature search.

Comprehensive and locally responsive legislation empowers local governments to advance and stand by the law and execute their obligations and capacities straightforwardly and genuinely. I reviewed the existing pieces of literature to explore if there have been any previous studies on this topic, particularly in the context of the Federal Republic of Nepal. Nepal (Fig. 1). However, although numerous legislations have been enacted by different local rural municipalities to make good governance a reality in the form of desired improved welfare programs, better management of health and educational services, and better delivery of products and services [[30], [31], [32]], there is a paucity of knowledge regarding the performance of Nepalese local government in formulating the related laws to deliver the rights assured by the constitution of Nepal.

The constitution of Nepal 2015 has granted the local government the full authority to formulate and enact laws. The executive, the municipal assembly, and the legislative committee are the three organs of the local government that are involved in making laws. At first, the executive body prepares legislative proposals which are then discussed in the legislative committee. After discussing the proposed law, the legislative committee drafts the initial proposal. The initial draft passed by the legislative committee is then discussed in the executive body. Following the approval of the proposed law by the executive body, it is presented to the municipal assembly for final approval. When the law is approved by the municipal assembly, the executive develops rules, guidelines, and other necessary measures based on that act.

Presently, there are 753 local governments across the 77 districts, a small but specific governing unit to administer local affairs. Among them, Kaski district, the headquarters district of Gandaki province has five local governmental units including one Metropolitan City and four rural municipalities. Within the last few years, the five local units under study have promulgated several legislations and directives, with a focus on fundamental laws and activities essential for their customary working and giving administrations, services, and mainly the development of the local people of the region. The existing status and the law-making processes in local governments of five local units of Kaski district have been varied and an erudite learning process [33]. Hence, this study aims to assess the laws made by the local levels of the Kaski district concerning the constitutionally affirmed 22 local-level powers (jurisdiction). Here, we specifically raised the following questions: (I) Did the five local levels of Kaski district over the last five-year period formulate the essential laws related to the conveyance of constitutionally endorsed 22 local-level powers? If not (ii) how many laws were formulated during the first five years' period and what are their specific objectives? (iii) What laws are yet to be made to execute the 22 local-level powers? The findings of this study thus shed light on the competencies of local governments in laying the foundation for the functioning of local government by formulating local laws on providing services to the people and also the complications faced by the legislative body while formulating the laws. Extending the findings beyond the case study, the findings of this study thus provide baseline information on Nepal's experience in implementing the federal government system.

## 2 Methodology

### 2.1 Study sites

**Fig. 2:**
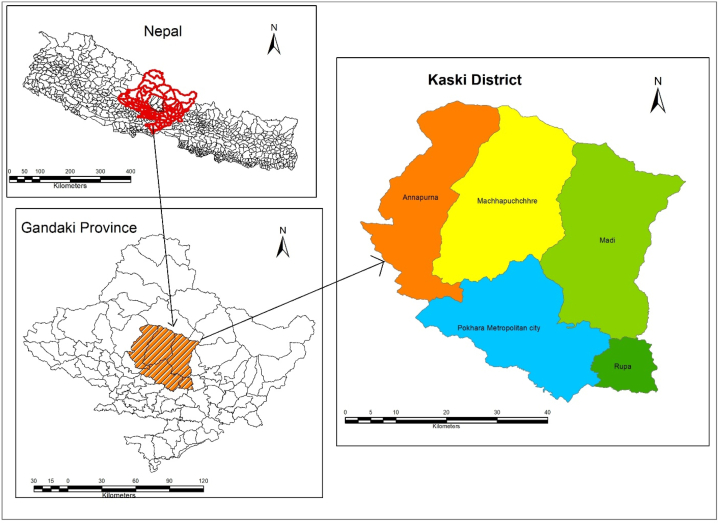
Location map of study sites.

Source: Department of Survey, Nepal http://dos.gov.np/

The study was conducted in the five local governments of Kaski District: Pokhara Metropolitan City, Annapurna Rural Municipality, Machhapuchre Rural Municipality, Madi Rural Municipality, and Rupa Rural Municipality. Pokhara Metropolitan City is the headquarters of Kaski District as well as the capital of Gandaki Province. The geographical position of the study sites within the country and the provincial context is presented in Fig. 2 [34]. A brief introduction to five local levels of the Kaski district is presented below.

Pokhara Metropolitan City, the largest metropolitan city of Nepal in terms of area, was established in May 2017. This metropolitan city constitutes 33 wards with a total population of 513,504 [35]. There are 170 council members and 43 executive members in Pokhara Metropolitan City [36].

Annapurna Rural Municipality has 11 wards and is extended over an area of 417.74 square kilometres [37], with a population of 22,099 [38]. There are fifty-nine council members and nineteen executive members in Annapurna Rural Municipality.

Machhapuchhre Rural Municipality has 9 wards with a total area of 545 square kilometers [39], and a population of 22,898 [40]. This rural municipality consists of 49 council members and 17 executive members.

The Madi Rural Municipality has 12 total wards and is extended over an area of 563 square kilometres [41], with a population of 16,142 [42]. This rural municipality has 64 council members and 20 executive members.

Rupa Rural Municipality has 7 wards with an area of 94.81 square kilometers [43], and a population of 14,891 [44]. This rural municipality has 39 council members and 15 executive members.

### 2.2 Methods of data collection

I conducted a questionnaire and interview survey with the key respondents of the respective local governmental bodies. From each local governmental body altogether 21 respondents were selected using a purposive and random sampling strategy. The Mayor/the Chairman, Vice-Mayor/Vice-Chairman, and the executive officer from each of the five local levels were purposely selected for the interview/questionnaire survey while the other 18 respondents were selected by random sampling method. The respondents of the latter group were selected in such a way to include six members from each of the various groups including ward chairmen, ward women representatives, and ward general members of the respective local governmental body. A set of questionnaires [Table S1] consisting of open-ended questions was asked to each of the respondents. Because the respondents are likely to be unfamiliar with the questions, and there could be a chance that information would be reported falsely, we provided a general review of the tool's sections and a succinct explanation of the study's purpose before the data collection. To make the interview convenient, the questionnaire was translated into Nepali, and the interviews/surveys were taken at their home/office. The interviews/questionnaire surveys were conducted from April 10 to April 30, 2022.

## 3 Results

### 3.1 Pokhara Metropolitan City

This metropolitan city enacted 98 laws, such as economic law, health law, cooperative law, good governance law, education law, municipal police law, etc. In the first year, altogether 21 laws (acts, by-laws, regulations, directives, procedures, and policies) were made. In the second, third, fourth, and fifth years, respectively 14, 13, 22, and 5 different laws were formulated by the council. These laws were formulated to facilitate and govern the day-to-day activities of residents. These laws are directly related to the general public. The ten most important laws related to the daily activities of people and their goals and implications are presented in Table 2 while the rest of the laws formulated by the metropolitan city are listed in a supplementary file [Supplementary file: Table S2]. Similarly, the executive of the Pokhara Metropolitan has made different procedures, directives, and rules for the easy implementation of laws made by the council. A few respondents’ opinions were as follows:

**Table 2:** Ten most important laws made by Pokhara Metropolitan City during the first five years [2017–2022] of operation.

S.N	Name of laws	Objectives	Constitutional Local-Level Powers
1	Market Monitoring Guide 2074	Quality control	Schedule-8, No. 10
2	Women program management procedure with deputy head 2074	Empowerment of women	Article 38
3	Public Private Partnership Act, 2074	Increase private participation in infrastructure building	Schedule-8, No. 7
4	Cooperative Regulations 2074	Income generation	Schedule-8, No. 2
5	Education Regulations 2074	To improve the quality of school-level education	Schedule-8, No. 8
6	Health Policy 2074	To improve public health	Schedule-8, No. 9
7	Pokhara Metropolitan Municipality Governance Act 2075	Well governance	Article 214
8	Vocational Education Act, 2076	Empower skill manpower	Schedule-8, No. 5, 17
9	Judicial Committee Act 2074	Systematize the work of the judicial committee	Article 217
10	Urban Planning Commission Formation and Operation Regulations 2074	Managing urbanization and improving the quality of living	Schedule-8, No. 7

The laws were designed to promote local growth and good administration. In the course of an interview, the Vice Chairman argues that laws were also created to empower women, allowing them to invest in small-scale cottage industries for their livelihood and give soft employment for their skill development. Ward number 16 chairman another respondent view is that federal government secretariat (Shinghadarbar) rights were delivered from the Pokhara ward office in less than 20 min; this is excellent practice for citizenship, individual birth, death, and marriage registrations; it also demonstrates our commitment to open government. The main objective is to raise human consciousness. According to a responder from Ward number 5, the Dalits and women do not meaningfully engage in the formulation of laws, and it is not discussed if the laws would be advantageous to the community as a whole. There is also little discussion of the significance of having sovereign power chosen by the people. Environmental preservation for park construction and sanitation, landscaping along highways, and effects on Pokhara Metropolitan City. We have established the foundation for a smart city, from the perspective of one respondent, ward number 13 chairman, by creating a digital ward charter with 13 no-ward offices to make it simple for residents and management to access services. Closed-circuit television (CCTV) is utilized to manage the local communities' security in this ward.

### 3.2 Annapurna Rural Municipality

As per the provisions of the constitution, the Annapurna Rural Municipality has adopted key acts, regulations, operating instructions, and codes of behaviour [45]. The Annapurna rural municipality has created 34 significant laws, regulations, operational instructions, and codes of conduct under the provisions of the constitution. In the last five years, it has produced 20 acts, 2 rules, 9 codes of conduct, and 3 directives. The municipality created the Financial Act 2018, Judicial Code of Conduct 2017, Local Health Sanitation Act 2020, Natural Resource and Conservation Act 2020, and Education Regulation 2017. In a similar vein, codes of conduct and procedures for infrastructure development have also been published. Table 3 lists the objectives and implications of these laws, which are directly related to the general population. While a supplementary file [Table S3] lists the remaining laws. Some respondents’ views are presented below:

**Table 3:** Ten most important laws made by Annapurna Rural Municipality during the first five years [2017–2022] of operation.

S.N	Name of laws	Objectives	Constitutional Local-Level Powers
1	F. M. Radio operation and Management Procedure, 2074	To register and monitor F. M. at the local level	Schedule-8, No. 2
2	Directives for Market Monitoring, 2074	To protect consumer rights	Schedule-8, No. 10
3	Public Private Partnership Act, 2077	Increase private participation in infrastructure building	Schedule-8, No. 7
4	Cooperative Act, 2074	To register, regulate, and monitor local cooperatives	Schedule-8, No. 2
5	Environment and Natural Resources Protection Act, 2077	Protecting the environment and natural resources	Schedule-8, No. 10, 21
6	Health and Sanitation Act, 2077	To improve public health	Schedule-8, No. 9
7	Disaster Risk Reduction and Management Act, 2077	Institutional Preparation for disaster risk reduction and response to minimize impact	Schedule-8, No. 20
8	Education Regulation, 2074	To improve the quality of school-level education	Schedule-8, No. 8
9	Procedure regarding Judicial Committee, 2074	Systematize the work of the judicial committee	Article 217
10	‘D' Class Construction License Distribution Procedure, 2074	Empowerment local construction	Schedule-8, No. 2, 11

The vice-chairman's vision opinion is “Our election manifesto promises the building of roads and bridges for people in need, as well as one water tap per residence. To guarantee that the general public receives goods and services, popular laws must have been approved by this point. Our first objective is development, and the people have granted us the power to adopt a variety of laws. In a similar vein, we wish to make agricultural goods but are unable to do so due to a lack of sufficient budget and technology. Grants are given to farmers to boost the agricultural output and the means of subsistence in the area. Ward Number 2 Chairman's standpoint is that: Through our timely and excellent service to the home centre, we have given them good governance, and the ordinary person is happy to receive it. The local administration of Annapurna Municipality Dhikurpokhari created municipal hospitals and health posts in each ward to take care of people's health. This represents the pinnacle of local achievement and outstanding service using more local vehicles than necessary, neglecting to take into account the populace, and collecting additional taxes from individuals who are already facing daily financial difficulties due to unemployment or other sources of income.

### 3.3 Machhapuchhre Rural Municipality

Machhapuchhre rural municipality, just like other local bodies of the Kaski district, was effortful in the formulation of local laws within the given constraints of the available manpower, resources, and budget. They lack qualified staff even if they have training and experience. As local laws, policies, and work plans form the cornerstone of local government, Machhapuchhre Rural Municipality has passed 54 laws in the past five years to promote good governance and general growth [46]. Those legislations include 12 acts, 5 regulations, 32 procedural laws, 4 directives, and 1 code of conduct. These regulations affect how locals conduct their daily lives. The goals and effects of these laws are listed in Table 4, many of which directly affect people in general. The remaining legislation is listed in a supplementary file [Table S4]. Broadly, this municipality is stimulating local infrastructure development, decent governance, and overall development of the common public. The Magar community in the Machhapuchhre Rural Municipality teaches their native tongue in primary schools. The provision of mother tongue language education and course design in the constitution is the most notable local-level accomplishment.

**Table 4:** Ten most important laws made by Machhapuchhre Municipality during the first five years [2017–2022] of operation.

S.N	Name of laws	Objectives	Constitutional Local-Level Powers
1	Market Monitoring Guide 2074	Quality control	Schedule-8, No. 10
2	Machhapuchare Rural Municipality Natural Resources Protection and Management Act, 2076	Machhapuchare Rural Municipality Natural Resources Protection and Management Ac	Schedule-8, No. 10,21
3	Public Private Partnership Act, 2074	Increase private participation in infrastructure building	Schedule-8, No. 7
4	Machhapuchare Rural Municipality Cooperative Act, 2074	Income generation	Schedule-8, No. 2
5	Education Act, 2075	To improve the quality of school-level education	Schedule-8, No. 8
6	Machhapuchre Health and Sanitation Act 2075	To improve public health	Schedule-8, No. 9
7	Machhapuchare Rural Municipality Natural Resources Protection and Management Act, 2076	Protecting the environment and natural resources	Schedule-8, No. 10,21
8	Machhapuchare Rural Municipality Procedure on the operation of Program on Pregnant Women Safety Program with Vice President, 2077	Women's Development and Children' development	Schedule-8, No. 5
9	An act relating to the Procedure that the Judicial Committee has to follow regarding hearing of complaints, 2075	Systematize the work of the judicial committee	Article 217
10	Scholarship fund Operation Procedure, 2077	To provide scholarships to talented and economically poor students	Schedule 8, No.

According to a respondent from Ward number 5, legislation protecting animals and birds has been passed, and our ecosystem has been protected. We have picked out a place for the vultures, and they also have a restaurant there. Our society also gathers older animals, where vultures are protected from dying by starvation. We donated the gift to protect it. Vice Chairmen's argument: In this municipality, the Magar community teaches their native dialect at the primary school. The most important local-level achievement is the provision for mother tongue language education and course design in the constitution.

### 3.4 Madi Rural Municipality

A total of 88 laws, including 22 acts, 8 regulations, 53 procedural laws, 3 directives, and one code of conduct, have been passed by the Madi Rural Municipality Council in just five years [47]. These pieces of legislation include laws in those areas that are within the jurisdiction of the local level as provisioned by the constitution. Table 5 lists the objectives and outcomes of various laws, many of which have a direct impact on society as a whole. A supplementary file contains a list of the remaining laws [Table S5].

**Table 5:** Ten most important laws made by Madi Rural Municipality during the first five years [2017–2022] of operation.

S. N.	Name of laws	Objectives	Constitutional local-level powers
1	Madi Rural Municipality Education Regulation, 2075	Quality education	Schedule-8, No. 8
2	Madi Rural Municipality Social Reform and Cost Efficiency Procedure, 2075	Justice	Association Registration Act, Section 4
3	Madi Rural Municipality Physical Infrastructure Management Act, 2075	Physical infrastructure development	Schedule-8, No. 11
4	Municipality Police Operation Procedure, 2076	Security	Schedule-8, No. 1
5	Madi Rural Municipality Physical Infrastructure Management Act, 2075	Physical infrastructure development	Schedule-8, No. 11
6	Procedure on Management of Drinking Water and Sanitation Consumer Organization, 2076	Health Care	Schedule-8, No. 7,21
7	Madi Rural Municipality Physical Infrastructure Management Act, 2075	Physical infrastructure development	Schedule-8, No. 11
8	Madi Rural Municipality Procedure relating to Selection and Recommendation of Teachers in Contract, 2077	Qualified teacher selection	Schedule-8, No. 8
9	Madi Rural Municipality Procedure relating to Distribution of Allowance for Fully Disabled People Caretaking, 2078	Justice	Schedule-8, No. 16
10	Madi Rural Municipality Procedure Relating to Operation of Pregnant Women Visiting Program, 2078	Health care	Schedule-8, No. 8

According to Section 64(2) of the Consumer Protection Act, the concerned Rural Municipality or Municipality may, to make the activities relating to market monitoring systematic and effective, frame and enforce necessary directives and procedures. Using the authority given by federal law, Madi rural municipality has framed such directives on consumer protection and market monitoring. Before the start of every fiscal year, Madi rural municipality enacts fiscal legislation; the Finance Act, and the Act relating to the allocation and expenditure of funds. The Finance Act can amend the existing tax rate which is within the scope of the local level like the wealth tax, house rent tax, land and building registration fee, land revenue, penalty, entertainment tax, and charges for local services. The views of some of the elected members of this municipality were as follows:

The Local Government Operation Act of 2017 has granted authority to local governments to collect money from the sale of stone, stones, sand, and other river goods. Similar to this, Environment Regulation 2020 stipulates that a preliminary environmental assessment must be done at the local level if the extraction of river-related products has a considerable negative impact on the immediate environment and surroundings. The following reply ward chairman suggests that the local levels have the authority to create and carry out environmental and public health legislation. Another woman who responded said that rural women benefited from cooperatives because there is only one cooperative member per household.

### 3.5 Rupa Rural Municipality

Rupa Rural Municipality has contributed to the creation of necessary local laws, just like the other rural municipalities in the Kaski district. Over five years, the local legislatures and governors have passed several different sorts of laws in the rural municipality of Rupa. There are 19 acts, 1 policy, 4 rules, 22 procedural rules, 6 directives, and 4 standards among the laws. It is a recent development in the community [48]. So far, a total of 56 laws have been passed by the Rupa Rural Municipality Council in the five years. The objectives and results of many laws are listed in Table 6, many of which have an immediate effect on society as a whole. A list of the remaining statutes can be found in a supplementary file [Table S6]. Effective market management has created day-to-day implications for laws that safeguard consumer rights. Education for enabling good knowledge and health care. Similar cooperatives help the locals support their way of life. They streamlined assistance and support for those who are disabled and created a law to safeguard the environment and promote good health. Rupa rural municipality has enacted procedural laws relating to the functioning of the Judicial Committee but, other than this, till now no other judicial bodies or dispute settlement mechanisms have been set up nor have laws relating to them been enacted. The chairman of the Rupa rural municipality argues that the rural municipality is working to ensure social and economic justice for the people and is fully accountable to the public while formulating laws.

**Table 6:** Ten most important laws made by Rupa Rural Municipality during the first five years [2017–2022] of operation.

S.N	Name of laws	Objectives	Constitutional Local-Level Powers
1	Rupa Rural Municipality Directives for Market Monitoring, 2075	To protect consumer rights	Schedule-8, No. 10
2	Rupa Rural Municipality Directives for Market Monitoring, 2075	To protect consumer rights	Schedule-8, No. 10
3	Rupa Rural Municipality Directives relating to the distribution of Identity Cards to People with Disabilities, 2075	Helps to provide additional facilities from the local level to people with disabilities	Schedule-8, No. 16
4	Rupa Rural Municipality Cooperative Act, 2077	Regulation of Cooperatives	Schedule-8, No. 2
5	Rupa Rural Municipality Procedure relating to joint program with teachers on one teacher one laptop handover program for promoting technology in education, 2077	Improving the quality of education and motivating teachers	Schedule-8, No. 8
6	Rupa Rural Municipality Health and Sanitation Act, 2075	To protect public health and maintain surrounding clean and hygienic	Rupa Rural Municipality Health and Sanitation Act, 2075
7	Rupa Rural Municipality Health and Sanitation Act, 2075	To protect public health and maintain surrounding clean and hygienic	Schedule-8, No. 9
8	Rupa Rural Municipality Directives relating to the distribution of Identity Cards to People with Disabilities, 2075	Helps to provide additional facilities from the local level to people with disabilities	Schedule-8, No. 16
9	Code of conduct for officials of Rupa Rural Municipality, 2074	To maintain good governance and discipline	Article 218
10	Rupa Rural Municipality Procedure relating to Brief Environmental Study and Initial Environmental Examination, 2077	Protection of environment and natural resources	Schedule-8, No. 1

Table 7 presents a comparative analysis of the laws enacted by the five local governments of the Kaski district using other rights in addition to the 22 constitutionally granted rights in five years. Pokhara Metropolitan City has passed 98 pieces of legislation in under five years. There are 22 fundamental rights listed in Schedule 8 of the Constitution, and an additional 8 pieces of legislation are now in the works. Similar to this, the Annapurna rural municipality approved 34 pieces of legislation, one of which dealt with constitutional rights. Schedule 8 of the Constitution lists 22 constitutional rights, of which 8 rights lists have been made and 14 pieces of legislation are currently pending. Machhapuchre rural municipality has passed 54 pieces of legislation, one of which concerns constitutional rights. On Schedule 14 of the Constitution, there are 22 constitutional rights stated; eight of these rights lists have been made, and there are eight more pieces of legislation that are still in the works. One of the 88 articles of legislation passed by the Madi Rural Municipality concerns constitutional rights. Schedule 8 of the Constitution lists 22 constitutional rights, of which 18 lists have been made and 4 pieces of legislation are still pending. One of the 54 articles of legislation passed by the Rupa Rural Municipality concerns constitutional rights. Schedule 8 of the Constitution contains 22 constitutional rights, of which 10 rights lists have been made and 12 pieces of legislation are currently pending.

**Table 7:** Comparative study of law-making by five local governments of Kaski district.

SN	Municipality	22 Rights		Remarks
		Laws Made	Legislation Pending	
1	Pokhara Metropolitan City	14	8	3,6,11,12,13,14,17,19
2	Annapurna Rural Municipality	8	14	1,3,4,5,6,12,13,14,15,16,17,18,19,22
3	Machhapuchre Rural Municipality	14	8	1,3,6,12,13,14,17,19
4	Madi Rural Municipality	18	4	3,6,13,18
5	Rupa Rural Municipality	10	12	1,3,4,6,11,12,13,14,17,19,21,22

The five local governments of the Kaski district have so far created several laws that support efficient, unbiased, and high-quality daily supply of products and services to the community. These laws have established the framework for how local government would operate. These laws serve as the cornerstone for delivering services, achieving residents' ambitions for growth, and meeting the requirements of local self-governance.

## 4 Discussion

Municipalities are exercising all of their constitutional powers at the local level abiding by the requirements as prescribed. It is to be noted that the constitution of Nepal upholds the values and principles of participatory democracy and guarantees 22 exclusive functions in addition to other concurrent rights. The local level rights include providing public services and fostering the growth of mother tongues, local cultures, and festivals. Local governments under the current study implemented several strict rules following the norms and spirits of the Nepal Constitution, making it simpler for the newly elected people's representatives to oversee and manage local governance activities.

The findings reveal that local levels have enacted the most important legislation, work practices, directions, regulations, and standards despite a lack of expert availability. The majority of their legal documents were already in force because they had been released and made public in the national gazette. The formulation of various important laws has been delayed or stalled by the federal and provincial administrations, and this harms regional development initiatives. Although the elected officials have made an effort to behave more properly toward the public, it is unfortunate that they lack experience.

The federal democratic republican is being institutionalized as a result of the elected local representatives' efforts to adopt grassroots development as much as feasible. The elected officials, however, lack the specific knowledge necessary to draft effective laws that will last for a very long time and benefit future generations. Although the laws are a great local policy, individuals could not understand its standards and principles because of a lack of comprehension.

Local authorities have been in charge of local-level school administration under constitutional rights. Local government has so far created courses on key historical occurrences at the local level, contemplation on natural resources, and local language course design and implications [49]. For instance, the rural communities of Machhapuchre and Madi at the primary level of education use the Magar and Gurung languages.

Cooperative laws are created by local governments, and they have positive effects on all rural municipalities and large cities because they encourage participation and growth of economic activity among the most basic citizens. The provincial government is authorized under the constitution to decide how laws are made locally. By this constitutional clause, Gandaki Province passed legislation governing how local assemblies should operate. Procedures governing the operation of village assemblies and municipal assemblies may be adopted and put into effect at the local level, but they should not conflict with applicable federal and provincial laws. This procedural legislation can aid in the smooth operation of the village or municipal assembly and the carrying out of its decision. The Kaski district's local levels have created a huge variety of regulations, although it can be challenging to discover ones that substantially impact the lives of local people. A lot of these laws were passed only as formalities. A mechanism needs to be set up for the execution of numerous laws. The Social Behavior Reform Act of 1976 is an example of a law that should be framed at the local level and should serve to improve the quality of service delivery at the local level [50].

As of now, the local populace can easily access the daily government system and receive services from the ward office located close by, including personal registration, citizenship, and passport applications [51]. In this regard, ward offices serve as people representatives who are answerable to the people. They work to offer goods and services to the public in a way that is efficient, impartial, and of high quality. The ultimate objective of local government is to enhance local people's quality of life. However, despite spending enormous amounts of money, the development sector as a whole lacks quality. Broadly, it can be said that many works are to be done in the future requiring further research studies in this field of study. However, only five local units of the Kaski district of Gandaki province make up the research area. Hence the results of the study could not apply to the entirety of Nepal due to these limited study units.

## 5 Conclusions

The federal constitution of 2015 has given independent and authoritative status to the local governments within the jurisdiction. It represents a turning point in local governance. From the studies of five local governments of Kaski district, it was found that the local governments have laid a foundation for the functioning of local-level government by formulating local laws in the first five years of their establishment. These local governments have formulated local laws of mainly two kinds. The first kind of laws are more or less similar and are mandatory laws, as they provide a legal set-up for all local governments to function administratively. Laws like municipal operating procedures, procedures related to conducting the meeting of the municipal executive, finance act, appropriation act, etc. belong to the laws of the first kind. The second kind of law addresses the distinctive needs of the particular local government so that they can provide service delivery, meet the distinctive development aspirations of the local people, and fulfil the needs of local self-governance. Laws like Agriculture Market Directives 2020, and scholarship distribution procedures for private Schools 2018 formulated by Pokhara Metropolitan belong to the laws of the second kind which also include a few other additional regulations like Smart City, youths’ sports, women empowerment, etc.

Each local unit under this study has passed an average of seventy laws that have an impact on its overall development as well as the efficient supply of high-quality services to the people. Of course, local governments have smaller budgets than the provincial or federal, but they have used a participatory development model in the law-making and development process. Although local governments are working to formulate laws for their local needs, the process of law formulation has been largely constrained by the lack of expertise in law-making, lack of resources, and budget. Despite several challenges in formulating laws, they have so far been successful in formulating basic mandatory laws and laws of a certain distinctive nature. This has shown gradual progress in the law-making capacity of the local governments and a strong sign of local self-governance as envisioned by the constitution of 2015. The accomplishments achieved so far are satisfactory and pave a strong foundation for further works in the law-making process in the local governments.

### Ethics statements

Before conducting a questionnaire/interview survey, I obtained research permission from the executive bodies of the five local governments of the Kaski district, i.e. the executive body of Pokhara Metropolitan City, Annapurna Rural Municipality, Machhapuchre Rural Municipality, Madi Rural Municipality, and Rupa Rural Municipality provided research permission (Permission Approval Number: 969) for the current study. Free prior and informed consent [FPIC] was taken from all the respondents. The respondents were also informed that the collected information would be used only for this research purpose and their identity would not be disclosed elsewhere.

## Funding statement

This research did not receive any specific grant from funding agencies in the public, commercial, or not-for-profit sectors.

## Data availability statement

All the data have been directly included in the main text/the supplementary file.

## CRediT authorship contribution statement

**Girdhari Dahal:** Writing – review & editing, Writing – original draft, Validation, Resources, Project administration, Methodology, Investigation, Formal analysis, Data curation, Conceptualization.

## Declaration of competing interest

The author declares that he has no known competing financial interests or personal relationships that could have appeared to influence the work reported in this paper.
