# Supplementary material for: Nepal's experience in implementing the federal government system: Assessment of law-making by the local governments of Kaski district, Nepal
Source: Heliyon. 2024 Feb 10;10(4):e26250. doi: 10.1016/j.heliyon.2024.e26250 (PMC10882041; doi:10.1016/j.heliyon.2024.e26250)
Supplement: Multimedia component 1 [file mmc1.docx]

**Nepal’s Experience in Implementing the Federal Government System: Assessment of Law-Making by the Local Governments of Kaski District, Nepal**

Girdhari Dahal^1*^

^1^Department of Political Sciences, Prithvi Narayan Campus, Tribhuvan University, Pokhara, Nepal

^*^For correspondence:[gddahal1234@gmail.com/](mailto:gddahal1234@gmail.com/)ORCID: [0000-0003-4287-098X](javascript:popup_orcidDetail(%22https://orcid.org%22,%20%220000-0003-4287-098X%22);)

**Supplementary File**

**Table S1: Questioners for the survey with the key respondents**

1. What laws are required by your local government's governance structure?
2. How many laws are made, and how are the locals held accountable?
3. Which requirement is most pressing at the local level?
4. How much was the local level right list provided?
5. How many laws were created throughout the first five years, and what were their particular goals?
6. What legislation must still be passed to carry out the 22 local-level powers?

| **Table S2: Laws made by Pokhara Metropolitan City during the first five years (2017-2022) of operation** | | | |
| --- | --- | --- | --- |
| S.N | **Name of laws** | **Objectives** | **Constitutional Local level Powers** |
| 1 | Municipal Assembly Operating Procedure 2074 | legalization decision | Article 214 |
| 2 | Economic Act 2074 | Budget formation | Article 228, 229 |
| 3 | Procedures related to conducting the meeting of the municipal executive 2074 | Monthly meeting legalization | Article 218 |
| 4 | Appropriation Act 2074 | Functioning | Article 229 |
| 5 | Performance Regulations 2074 | Quality maintain | Article 214 |
| 6 | Certification (Procedure) Niyamavali 2074 | Legalization | Article 218 |
| 7 | City Executive (Division of Work) Niyamali 2074 | Elected members power sharing | Article 218 |
| 8 | Judicial Committee (Regarding Procedure) Act 2074 | Systematize work of judicial committee | Article 217 |
| 9 | Market Monitoring Guide 2074 | Quality control | Schedule-8 , No. 10 |
| 10 | Act to regulate the rights of children 2074 | Security to the child | Article 39 |
| 11 | Garbage Management Regulations 2074 | Care of Health | Schedule- 8, No. 9 |
| 12 | Agricultural Business Promotion Act 2074 | Motivation to the people | Schedule- 8, No. 15, 18 |
| 13 | Agricultural policy 2074 | Improvisation in agriculture production and use of modern technology in agriculture | Schedule- 8, No. 15, 18 |
| 14 | Women program management procedure with deputy head 2074 | Empowerment of women | Article 38 |
| 15 | Animal husbandry policy 2074 | Management of animals | Schedule- 8, No. 15 |
| 16 | Public Private Partnership Act, 2074 | Increase private participation in infrastructure building | Schedule-8 , No. 7 |
| 17 | Public Private Partnership Policy 2074 | Increase private participation in infrastructure building | Schedule-8 , No. 7 |
| 18 | Policy to regulate advertisement boards and promotional materials 2074 | To maintain city beauty, to regulate market |  |
| 19 | Cooperative Regulations 2074 | Income generation | Schedule-8, No. 2 |
| 20 | Education Regulations 2074 | To improve quality of school level education | Schedule-8, No. 8 |
| 21 | Health Policy 2074 | To improve public health | Schedule-8, No. 9 |
| 22 | Urban Planning Commission Formation and Operation Regulations 2074 | Long term planning and systematic urbanization | Schedule-8, No. 7 |
| 23 | Disaster Management Act, 2074 | Pre planning for disaster reduction and carry out immediate rescue after disaster | Schedule-8, No. 20 |
| 24 | Pokhara Municipal Corporation Cooperative Act | Income generation | Schedule-8, No. 2 |
| 25 | Pokhara Metropolitan Municipality Sports Development Act 2075 | Promoting sport activities for encouraging people to maintain physical fitness | Schedule-8, No. 8 |
| 26 | Pokhara Metropolitan City Executive Code of Conduct 2075 | Good management | Article 218 |
| 27 | Code of Conduct of Pokhara Metropolitan Municipality Officials 2075 | Well management for office | Article 214, 218 |
| 28 | Pokhara Metropolitan Municipality Tourism Development Act 2075 | Promote the tourism | Schedule-8, No. 4, 22 |
| 29 | Pokhara Metropolitan Municipality Financial Act 2075 (Amendment) | Budget mobilization, good governance promotion | Article 228, 229 |
| 31 | Economic Procedures Act of Pokhara Metropolitan Municipality 2075 | Budget mobilization, good governance promotion | Article 228, 229 |
| 32 | Pokhara Metropolitan City Police Management Act 2075 | Security | Schedule-8, No.1 |
| 33 | Act 2075 to manage infrastructure development of Pokhara Metropolitan City | Development | Schedule-8, No.7 |
| 34 | Pokhara Metropolitan Municipality Governance Act 2075 | Well governance | Article 214 |
| 35 | Pokhara Metropolitan Municipality Economic Act 2076 | Budget mobilization, good governance promotion | Article 228, 229 |
| 36 | Vocational Education Act, 2076 | Empower skill man power | Schedule-8, No.5, 17 |
| 37 | Act 2074 (First Amendment) 2076 to regulate public private partnership | Improvement PPP model | Schedule-8, No.7 |
| 38 | Animal Health and Animal Services Act 2076 | Secure animals | Schedule-8, No. 8, 15 |
| 39 | PokharaPragya Academy Act 2076 | Systematize indigenous knowledge | Schedule-8, No.22 |
| 40 | Public Transportation Act 2076 | Reform transportation | Schedule-8, No.7 |
| 41 | Personnel Service Act | To improve service delivery | Article 214, Schedule-8, No.5 |
| 42 | Judicial Committee Act 2074 (First Amendment) | Systematize work of judicial committee | Article 217 |
| 43 | Procurement Regulations 2076 | Supply of necessary goods | Article 228 |
| 44 | Building Regulation and Documentation Procedure 2076 | To fix standard of construction work |  |
| 45 | Urban Planning Commission Formation and Operation Regulations 2074 (First Amendment) 2076 | Managing urbanization and improve quality of living | Schedule-8, No. 7 |
| 46 | Economic Act 2077 | Regulation of all program | Article 228, 229 |
| 47 | Appropriation Act 2077 | To issue fund out of local consolidated fund | Article 229 |
| 48 | Tax and Non-Tax Revenue Act 2077 | Collection of revenue | Article 228, 229 |
| 49 | Tourism Development Act, 2077 | To systematize and facilitate tourism sector | Schedule- 8, No.4, 22 |
| 50 | Disaster Risk Reduction and Management Act, 2077 | Institutional Preparation for disaster risk reduction and response to minimize impact | Schedule-8, No. 20 |
| 51 | Environment and Natural Resources Protection Act, 2077 | Protecting environment and natural resources | Schedule-8, No. 10, 21 |
| 52 | Agriculture Act, 2077 | Promoting agricultural activities | Schedule-8, No. 15, 18 |
| 53 | Pokhara Metropolitan City Development Corporation Act, 2077 | Setting Institutional structure for city development | Schedule-8, No. 7 |
| 54 | Economic Procedure Act of Pokhara Metropolitan City 2075 (First Amendment) | Regulate budget use and bring transparency and efficiency in use of resources | Article 228, 229 |
| 55 | Gandaki Polytechnic Education Act, 2076 (First Amendment) | Establish polytechnic education institution | Schedule-8, No. 8 |
| 56 | Tax and Non-Tax Revenue Act 2077 (First Amendment) | Regulate budget use and bring transparency and efficiency in use of resources | Article 228, 229 |
| 57 | Pokhara Metropolitan Municipality Sports Development Act 2075 (First Amendment) | Promoting sport activities for encouraging people to maintain physical fitness | Schedule-8, No. 8 |
| 58 | Bill 2074 (Second Amendment) made to regulate public private partnership | Increase private participation in infrastructure building | Schedule-8 , No. 7 |
| 59 |  |  |  |
| 60 | Act 2075 (First Amendment) made to manage the infrastructure development of Pokhara Metropolitan | To bring quality in building infrastructure and manage infrastructure to facilitate larger group of people | Schedule-8 , No. 7 |
| 61 | Agricultural Business Promotion Act 2074 (First Amendment) | To promote agricultural activity and provide encouragement to farmers | Schedule-8 , No. 15, 18 |
| 62 | Pokhara Metropolitan Corporation Cooperative Act (First Amendment) | To register and regulate cooperatives established in Pokhara Metropolitan municipality | Schedule-8 , No. 2 |
| 63 | Pokhara Metropolitan Municipality Administration Act 2075 (First Amendment) | Improve Institutional structure and bring clarity in functioning | Article 214 |
| 64 | Division of Work Rules (First Amendment) | Bring clarity to exercise of jurisdiction | Article 218 |
| 65 | Performance Regulations (First Amendment) | To monitor the progress of budget utilization | Article 214 |
| 66 | Certification of City Executive Decisions and Powers of Attorney (First Amendment) | Procedural law relating to authentication of decisions of local level executive | Article 218 |
| 67 | Municipal Police Act (First Amendment) | To give clarity to functions and facilities to Metropolitan Police | Schedule-8, No. 1 |
| 68 | Second Amendment to the Constitution of the Urban Planning Commission | To make Urban Planning Commission active and effective | Schedule-8 , No. 7 |
| 69 | Pokhara Municipality Judicial Committee (Procedure ) 2074 Second Amendment | Procedural clarity to functioning of Judicial Committee | Article 217 |
| 70 | Pokhara Metropolitan Municipality Garbage Management Act 2074 First Amendment | Management of garbage | Schedule-8 , No. 9 |
| 71 | Pokhara Metropolitan Municipality Rights Act 2074 First Amendment Bill 2077 | Promotion of Child Right | Article 39 |
| 72 | Guidelines for training, seminars, workshops and study tour expenses 2074 | Procedural law relating to managing and proper use of funds |  |
| 73 | Legislation Committee Rule 2074 | Procedural clarity to law making | Article 226 |
| 74 | Procedure related to local gazette publication 2074 | Publication of laws and decisions of local level and increasing transparency | Article 214, 218 |
| 75 | Directory of Energy Development, 2075 | Provide scopes for energy development | Schedule-8, No. 19 |
| 76 | Pokhara Metropolitan Municipality's non-formal education teaching support management guide 2075 | Help to build skills | Schedule-8, No. 5, 8 |
| 78 | Public Private Partnership Regulations 2075 | To run different projects under collaboration and financial participation of both public and private sector |  |
| 89 | City Youth Council Operating Procedure 2075 | Formation and operation of youth council | Schedule-8 , No. 5 |
| 80 | Pokhara Metropolitan Municipality identity card distribution procedure for persons with disabilities | Procedural law relating distribution of different identity cards to disable people | Schedule-8, No. 16 |
| 81 | Necessary rules made regarding standards of Pokhara Metropolitan Municipality | Fixing standard and bringing uniformity in works | Schedule-8, No. 10 |
| 82 | Municipal Youth Council Operating Procedure 2075 (First Amendment) | Formation and operation of youth council | Schedule-8 , No. 5 |
| 83 | 'D" Class Construction Business License Procedure 2075 (First Amendment) | Registration and monitoring construction work |  |
| 84 | Procedures related to health institution registration, renewal, expansion and service addition in Pokhara | Registration and monitoring of health institution | Schedule-8 , No. 9 |
| 85 | Metropolitan City 2075 | Administrative improvisation and systematization | Article 214, 218 |
| 86 | Pokhara Metropolitan City Smart City Haw Project Operation Procedure 2075 | Use of information technology in city development | Schedule-8 , No. 7 |
| 87 | Pokhara Metropolitan City Toll Development Organization Operating Procedure 2075 | Systematize operation of Toll Development organizations | Schedule-8 , No. 5 |
| 88 | Animal Development Subsidy Procedure of Pokhara Metropolitan Municipality 2075 | Uniformity in distribution of subsidies to farmers | Schedule-8 , No. 15 |
| 89 | Pokhara Metropolitan Municipality Hospital Management Committee Formation Guidelines 2075 | Formation of Hospital Management Committee | Schedule-8 , No. 9 |
| 90 | Pokhara Metropolitan Municipality Audit Committee Procedure 207 | Procedural clarity for auditing | Article 229 |
| 91 | Pokhara Metropolitan City Mediation Center Operation Procedure, 2078 | Arbitration | Article 217 |
| 92 | Pokhara Metropolitan City Birthplace Prize Distribution Procedure, 2076 | Motivation | Schedule-8 , No. 5 |
| 93 | Pokhara Metropolitan City Procedure Related to Land Acquisition | Production |  |
| 94 | Working Procedure relating to functioning of Pokhara Metropolitan City Monitoring and Supervision Committee, 2078 | To assure consumer right and assure quality of goods and services | Article 214 |
| 95 | Pokhara Metropolitan City Youth Council Regulation Procedure, 2078 | Formation and operation of youth council | Schedule-8 , No. 5 |
| 96 | Pokhara Metropolitan City Work Operation Guidance, 2078 | To bring systematization in the functioning of local level | Article 214 |
| 97 | Pokhara Metropolitan City's Organization Registration and renew Procedure, 2078 | To register organization and renew them | Associations Registration Act, 2034, Section 4 |
| 98 | Procedure relating to formation and operation of Pokhara Metropolitan City Local Children's Club, 2078 | To empower children's and to listen their voice in public policy making | Children's Act, 2018, Section 58 |

| **Table S3: Laws made by Annapurna Rural Municipality during the first five years (2017-2022) of operation** | | | |
| --- | --- | --- | --- |
| **S. N** | **Name of laws** | **Objectives** | **Constitutional Local level Powers** |
| 1-5 | Finance Act, 2074,  Finance Act, 2075,  Finance Act, 2076,  Finance Act, 2077  Finance Act, 2078 | Budget formation | Article 228, 229 |
| 6-10 | Appropriation Act, 2074  Appropriation Act, 2075,  Appropriation Act, 2076  Appropriation Act, 2077  Appropriation Act, 2078 | To mobilize budget and use fund out of consolidated funds | Article 229 |
| 11 | Cooperative Act, 2074 | To register, regulate and monitor local cooperatives | Schedule-8 , No. 2 |
| 12 | Administrative Procedure Regulation Act, 2075 | To solve administrative problems relating to budget mobilization | Article 214 (3) |
| 13 | Work Division Regulation, 2074 | To make division of authority among different committees of Village Municipal executive | Article 218 |
| 14 | Procedure regarding Judicial Committee, 2074 | Systematize work of judicial committee | Article 217 |
| 15 | Education Regulation, 2074 | To improve quality of school level education | Schedule-8 , No. 8 |
| 16 | Village Assembly Operation Procedure, 2074 | To operate meeting of village assembly in systematic way | Article 226 |
| 17 | 'D' Class Construction License Distribution Procedure , 2074 | Empowerment local construction | Schedule-8 , No. 2, 11 |
| 18 | F.M. Radio operation and Management Procedure, 2074 | To register and monitor F.M. in local level | Schedule-8 , No. 2 |
| 19 | Consumer Committee Formation, Mobilization and Management Procedure, 2074 |  | Schedule-8 , No. 7, 10 |
| 20 | Meeting Operation and Management Procedure, 2074 | To systematize meeting of Rural Municipality Executive Meeting | Article 218 |
| 21 | Directives relating to protection and utilization of Natural Resources, 2074 | To protect and utilize of natural resources for the benefit of present and future generation | Schedule-8, No. 21 |
| 22 | Local Gazettee Publication Procedure, 2074 | To make publication of local level decision and laws for the knowledge of public | Article 214 |
| 23 | Guidelines for training, seminars, workshops and study tour expenses 2074 | Procedural law relating to managing and proper use of funds | - |
| 24 | Procedure relating to Management of Technical Staff in Contract, 2074 |  | Article 214 |
| 25 | Directives for Market Monitoring, 2074 | To protect consumer rights | Schedule-8, No. 10 |
| 26 | Act relating to operation of program on Social security for orphan and vulnerable children's, 2077 | Helps in operation of program on Social security for orphan and vulnerable children's | Social Security Act, 2075, Section 27 |
| 27 | Economic Procedure Regulation Act, 2077 | Budget utilization and promotion of good governance | Article 228, 229 |
| 28 | Infrastructure Management Act, 2077 | To make infrastructure long lasting and bring quality in infrastructure construction | Schedule-8, No, 7 and 11 |
| 29 | Disaster Risk Reduction and Management Act, 2077 | Institutional Preparation for disaster risk reduction and response to minimize impact | Schedule-8 , No. 20 |
| 30 | Environment and Natural Resources Protection Act, 2077 | Protecting environment and natural resources | Schedule-8 , No. 10,21 |
| 31 | Public private partnership Act, 2077 | Increase private participation in infrastructure building | Schedule-8 , No. 7 |
| 32 | Local resources utilization and management Act, 2077 | To protect and utilize of local resources for the benefit of public | Schedule-8, No. 21 |
| 33 | Health and Sanitation Act, 2077 | To protect public health and maintain surrounding clean and hygienic | Schedule-8, No. 9 |
| 34 | Mediation Center Operation Procedure, 2078 | To promote alternative dispute resolution in civil cases | Article 217 |

| **Table S4: Laws made by Machhapuchhre Municipality during the first five years (2017-2022) of operation** | | | |
| --- | --- | --- | --- |
| **S. N** | **Name of laws** | **Objectives** | **Constitutional Local level Powers** |
| 1 | Machhapuchare Rural Municipality Finance Act, 2074 | Budget formation | Article 228, 229 |
| 2 | Machhapuchare Rural Municipality Appropriation Act, 2074 | Budget formation | Article 228, 229 |
| 3 | Machhapuchare Rural Municipality Cooperative Act, 2074 | To register, regulate and monitor local cooperatives | Schedule-8 , No. 2 |
| 4 | Act relating to Procedure that Judicial Committee has to follow regarding hearing of complain, 2075 | Systematize work of judicial committee | Article 217 |
| 5 | Machhapuchare Rural Municipality Disaster Risk Reduction and Management Act, 2075 | Institutional Preparation for disaster risk reduction and response to minimize impact | Schedule-8 , No. 20 |
| 6 | Machhapuchare Rural Municipality Natural Resources Protection and Management Act, 2076 | Protecting environment and natural resources | Schedule-8 , No. 10,21 |
| 7 | Machhapuchare Rural Municipality Physical Infrastructure Management Act, 2075 | To have long term planning and management of physical infrastructure development | Schedule-8 , No. 11 |
| 8 | Education Act, 2075 | Promote quality education in school level | Schedule-8 , No. 8 |
| 9 | Machhapuchare Rural Municipality Health and Sanitation Act, 2075 | To maintain health and Sanitation | Schedule-8 , No. 9 |
| 10 | Machhapuchare Rural Municipality Local Water Resources Act, 2077 | Protecting and proper use of water resources | Schedule-8 , No. 10,21 |
| 11 | Machhapuchare Rural Municipality Act relating to Youth Council Operation, 2077 | Participating youth in decision making and development activities | Schedule- 8 , No. 5 |
| 12 | Machhapuchare Rural Municipality Environment and Natural Resources Protection Act, 2078 | Protecting environment and natural resources | Schedule-8 , No. 10,21 |
| 13 | Machhapuchare Rural Municipalities Executive (Work Administration) Rule, 2074 | Systematize activities of executive | Article 218 |
| 14 | Machhapuchare Rural Municipality Executive (Work Division) Regulation, 2074 | Systematize activities of different committees of executive | Article 218 |
| 15 | Machhapuchare Rural Municipality Education Regulation, 2075 | To improved quality of school level education | Schedule-8 , No. 8 |
| 16 | Machhapuchare Rural Municipality Rule on Public Procurement | Promote good governance and economic transparency | Article 229 |
| 17 | Machhapuchare Rural Municipality Local Gazettee Publication Procedure, 2074 | To make publication of local level decision and laws for the knowledge of public | Article 214 |
| 18 | Machhapuchare Rural Municipalities Executives Meeting Operation and Management Procedure, 2074 | Systematize meeting of executive | Article 218 |
| 19 | Machhapuchare Rural Municipality Village Assembly Operation Procedure, 2074 | Systematize meeting of village Committee | Article 221 |
| 20 | Machhapuchare Rural Municipality Consumer Committee Formation, Mobilization and Management Procedure, 2074 | To involve local people in development activities | Schedule-8 , No. 7, 10 |
| 21 | Machhapuchare Rural Municipality Integrated tax Management Procedure, 2075 | Comprehensive | Schedule-8 , No. 4 |
| 22 | Machhapuchare Rural Municipality Procedure relating to distribution of Identity Cards to People with Disabilities, 2075 | Helps to provide additional facilities from local level to people with disabilities | Schedule-8 , No. 16 |
| 23 | Machhapuchare Rural Municipality Procedure relating to distribution of Identity Cards to Elderly Citizen , 2075 | Helps to provide additional facilities from local level to all elderly citizens living in the rural municipality | Schedule-8 , No. 16 |
| 24 | Machhapuchare Rural Municipality Procedure on Emergency Work Operation Center, 2075 | To manage service delivery during time of emergency | Schedule-8 , No. 20 |
| 25 | Machhapuchare Rural Municipality Natural Resources Protection and Management Act, 2076 | Protecting environment and natural resources | Schedule-8 , No. 10,21 |
| 26 | Procedure relating to formation and operation of Local Children's Club, 2075 | To empower children's and to listen their voice in public policy making | Children's Act, 2018, Section 58 |
| 27 | Procedure relating to Management of Machhapuchare Rural Municipality Technical Staff in Contract, 2074 | For administration of function of local level | Article 214 |
| 28 | Machhapuchare Rural Municipality D Class Construction License Distribution Procedure , 2075 | Bringing quality in construction work | Schedule-8 , No. 2, 11 |
| 29 | Procedure on Agricultural Network Program implementation, 2076 | Promoting agricultural activities | Schedule-8 , No. 15 |
| 30 | Procedure regulating to protection and promotion of the rights of children 2076 | Child right protection and promotion | Article 39 |
| 31 | Procedure relating to formation of Elderly Citizen Network , 2076 | Helps to provide additional facilities from local level to all elderly citizens living in the rural municipality | Schedule-8 , No. 16 |
| 32 | Procedure relating to formation and operation of Women Development Committee, 2076 | Protection of women's right and organizing program on women empowerment | Article 38 |
| 33 | Machhapuchare Rural Municipality Disaster Management Fund (Operation) Procedure, 2076 | Disaster management | Schedule-8 , No. 20 |
| 34 | Procedure relating to Brief Environmental Study and Initial Environmental Examination, 2077 | Protection of environment and natural resources | Schedule-8 , No. 21 |
| 35 | Directives on use of Chemical Fertilizer | To minimize adverse impact of chemical fertilizer | Schedule-8, No. 9, 15 |
| 36 | Machhapuchare Rural Municipality Procedure on establishment and operation of information and record Center, 2077 | To make publication of local level decision and laws for the knowledge of public | Article 214 |
| 37 | Procedure on operation of Program on distribution of grant to farmers involved in Animal husbandry for Milk Production , 2077 | To increase agricultural production | Schedule-8, No. 15, 18 |
| 38 | Scholarship fund Operation Procedure, 2077 | To provide scholarship to talent and economically poor students | Schedule 8, No. |
| 39 | Procedure on Management of Drinking Water and Sanitation Consumer Organization, 2077 | Drinking water management | Schedule-8 , No. 7,21 |
| 40 | Machhapuchare Rural Municipality Procedure on operation of Program on Pregnant Women Safety Program with Vice President, 2077 | Women's development and Children' development | Schedule-8 , No. 5 |
| 41 | Youth Self Employment Program (Youth Self Employment Fund) Operation Procedure, 2075 | To increase participation of youth in local affairs | Schedule-8 , No. 5 |
| 42 | Machhapuchare Rural Municipality Procedure on extraction, collection and sales of mineral resources from the Private Land, 2078 | To protect nature and environment and make wise use of natural resources | Schedule-8 , No. 5 |
| 43 | Machhapuchare Rural Municipality Procedure on operation of Gender Related Violence Reduction Fund, 2078 | To reduce gender related violence and promote social security | Article 38 |
| 44 | Machhapuchare Rural Municipality Procedure on operation of Gender Related Violence Reduction Fund, 2078 | To reduce gender related violence and promote social security | Article 38 |
| 45 | One education institution one Enterprise Program | To give vocational knowledge and teach methods of livelihood to students | Schedule-8 , No. 5, 8 |
| 46 | Machhapuchare Rural Municipality Procedure on operation of Insurance Coordination Committee | To promote the habit of doing insurance of health and property | Schedule-8 , No. 5 |
| 47 | Machhapuchare Rural Municipalities (Work Administration) Directives, 2074 | Systematize activities of Rural Municipality | Article 218 |
| 48 | Machhapuchare Rural Municipality Directives relating to Program Operation on Women and Children with Vice President, 2075 | Women's development and Children' development | Schedule-8 , No. 5 |
| 49 | Code of Conduct for Authorities of Machhapuchare Rural Municipality, 2074 | Maintain discipline among the elected representative and promote good governance | Article 214, Article |
| 50 | Infrastructure Management Act, 2077 | To make infrastructure long lasting and bring quality in infrastructure construction | Schedule-8, No, 7 and 11 |
| 51 | Disaster Risk Reduction and Management Act, 2077 | Institutional Preparation for disaster risk reduction and response to minimize impact | Schedule-8 , No. 20 |
| 52 | Environment and Natural Resources Protection Act, 2077 | Protecting environment and natural resources | Schedule-8 , No. 10,21 |
| 53 | Public private partnership Act, 2077 | Increase private participation in infrastructure building | Schedule-8 , No. 7 |
| 54 | Local resources utilization and management Act, 2077 | To protect and utilize of local resources for the benefit of public | Schedule-8, No. 21 |
| 55 | Health and Sanitation Act, 2077 | To protect public health and maintain surrounding clean and hygienic | Schedule-8, No. 9 |
| 56 | Mediation Center Operation Procedure, 2078 | To promote alternative dispute resolution in civil cases | Article 217 |

| **Table S5: Laws made by Madi Rural Municipality during the first five years(2017-2022) of operation** | | | | |
| --- | --- | --- | --- | --- |
| S.N | Name of laws | Objectives | Constitutional Local level Powers |  |
| 1 | Madi Rural Municipalities Act relating to Economic Procedure, 2075 | To collect revenue, to increase and decrease tax rate and manage fiscal administration | Article 229, |  |
| 2 | Madi Rural Municipality Administrative Procedure Regulation Act, 2075 | To solve administrative problems relating to budget mobilization | Article 214 (3) |  |
| 3 | Madi Rural Municipality Cooperative Act, 2074 | To register, regulate and monitor local cooperatives | Schedule-8 , No. 2 |  |
| 4 | Madi Rural Municipality Physical Infrastructure Management Act, 2075 | To have long term planning and management of physical infrastructure development | Schedule-8 , No. 11 |  |
| 5 | Act relating to Procedure to be followed while hearing and deciding complain by Judicial Committee, 2075 | To have uniformity in judicial function and to systematize work of Judicial Committee | Article 217 |  |
| 6 | Procedure relating to operation of program on Social security for orphan and vulnerable children's | Helps in operation of program on Social security for orphan and vulnerable children's | Social Security Act, 2075, Section 27 |  |
| 7 | Madi Rural Municipality Health and Sanitation Act, 2075 | To maintain health and Sanitation | Schedule-8 , No. 9 |  |
| 8 | Madi Rural Municipality local water resources Act, 2075 | Protection and utilization of water resources | Schedule-8 , No. 19 |  |
| 9 | Land utilization Act of Madi Rural Municipality, 2075 | Systematic division of land area into different category according to utility |  |  |
| 10 | Madi Rural Municipality Disaster Risk Reduction and Management Act, 2075 | Institutional Preparation for disaster risk reduction and response to minimize impact | Schedule-8 , No. 20 |  |
| 11 | Madi Rural Municipality Agriculture Enterprise Promotion Act, 2075 | Promoting agricultural activities | Schedule-8 , No. 15 |  |
| 12 | Primary and secondary Level Education Act, 2075 | Promote quality education in school level | Schedule-8 , No. 8 |  |
| 13 | Madi Rural Municipality Police Management Act, 2076 | To give clarity to functions and facilities to Metropolitan Police | Schedule-8 , No. 1 |  |
| 14 | Madi Rural Municipality Local Resources Utilization and Management Act, 2076 | Utilize and Manage Local Resources | Schedule-8 , No. 21 |  |
| 15 | Madi Rural Municipality Environment and Natural Resources Protection Act, 2076 | Protecting environment and natural resources | Schedule-8 , No. 10,21 |  |
| 16 | Madi Rural Municipality public private partnership Act, 2077 | Increase private participation in infrastructure building | Schedule-8 , No. 7 |  |
| 17 | Building Construction Standard, 2077 | Helps to maintain housing quality and build long term and strong structure buildings |  |  |
| 18 | Madi Rural Municipality Tax and non Tax Revenue Act, 2077 | Collection of revenue | Schedule-8 , No. 2 |  |
| 19 | Madi Rural Municipality Forest Area Protection and Management Act, 2078 | Protection of forest and its management | Schedule-8 , No. 10, 21 |  |
| 20 | Madi Rural Municipality Protection and promotion Children Right Act | Promotion of Child Right | Constitution of Nepal, Article 39 |  |
| 21 | Madi Rural Municipality Local Land Tenure Act, 2078 | Land management | Schedule-8 , No. 14 |  |
| 22 | Madi Rural Municipality Drinking Water, Cleanliness and Sanitation Act, 2078 | Awareness | Schedule-8 , No. 19 |  |
| 23 | Madi Rural Municipality Civil Servant Service Management Act, 2078 | Good Governance | Article 214 (3) |  |
| 24 | Madi Rural Municipality Executive (Function Administration) Regulation, 2074 | Day to day easy service delivery | Article 214 (3) |  |
| 25 | Madi Rural Municipality Executive (Work Division) Regulation, 2074 | Effective Service provide | Article 214 (3) |  |
| 26 | Code of Conduct for Authorities of Madi Rural Municipality, 2074 | Follow the rule of law | Article 214, Article |  |
| 27 | Madi Rural Municipalities Executives Meeting Operation and Management Procedure, 2074 | Good governance | Article 214 (3) |  |
| 28 | Ratification of Decision, Order and bill of rights issued by Madi Rural Municipality Executive, 2074 | Certify | Article 214 (3) |  |
| 29 | Madi Rural Municipality Village Assembly Operation Procedure, 2074 | Necessary law making | Article 221 |  |
| 30 | Madi Rural Municipality Physical Infrastructure Management Act, 2075 | To have long term planning and management of physical infrastructure development | Schedule-8 , No. 11 |  |
| 31 | Madi Rural Municipalities Toll Development Organization Operation Procedure, 2074 | Equal development |  |  |
| 32 | Madi Rural Municipality Economic Aid Procedure, 2074 | For general work | Article229 |  |
| 33 | Madi Rural Municipality Local Gazettee Publication Procedure, 2074 | Public notice | Article 214 |  |
| 34 | Procedure relating to Management of Madi Rural Municipality Technical Staff in Contract, 2074 | Technology friendly | Article214 |  |
| 35 | Madi Rural Municipality Consumer Committee Formation, Mobilization and Management Procedure, 2074 | Construction | Schedule-8 , No. 7, 10 |  |
| 36 | Madi Rural Municipality D Class Construction License Distribution Procedure , 2075 | legality | Schedule-8 , No. 2, 11 |  |
| 37 | Directives for Market Monitoring by Madi Rural Municipality , 2075 | Quality Service | Schedule-8 , No. 11 |  |
| 38 | Madi Rural Municipality Disaster Management Fund (Operation) Procedure, 2075 | Carful | Schedule-8 , No. 20 |  |
| 39 | Madi Rural Municipality Education Regulation, 2075 | Quality education | Schedule-8 , No. 8 |  |
| 40 | Madi Rural Municipality Program on Small Enterprise for Poverty Reduction Operation Procedure, 2075 | Use of skill | Schedule-8 , No. 5, 17 |  |
| 41 | Madi Rural Municipality Energy Development Directives, 2075 | Natural resource use | Schedule-8 , No. 19 |  |
| 42 | Madi Rural Municipality Non- Governmental Organization mobilization and management Procedure, 2075 | Private sector participation | Schedule-8 , No.5 |  |
| 43 | Madi Rural Municipality Enterprise and Profession Registration and Listing Procedure, 2075 | Quality service |  |  |
| 44 | Madi Rural Municipality Social Reform and Cost Efficiency Procedure, 2075 | Justice | Association Registration Act, Section 4 |  |
| 45 | Madi Rural Municipality Property tax Management Procedure, 2075 | Economic development | Schedule-8 , No. 4 |  |
| 46 | Madi Rural Municipality Procedure relating to Program Operation on Women and Children with Vice President, 2075 | Justice | Schedule-8 , No. 5 |  |
| 47 | Madi Rural Municipality Procedure on Operation of Program on Agricultural production based on Subsidies, 2075 | Motivation | Schedule-8 , No. 2, 15 |  |
| 48 | Madi Rural Municipality Procedure on Operation of Subsidies based Animal Development Program, 2075 | Motivation | Schedule-8 , No. 15 |  |
| 49 | Madi Rural Municipality President with Youth Self Employment Program (Youth Self Employment Fund) Operation Procedure, 2075 | Employment creation | Schedule-8 , No. 5 |  |
| 50 | Madi Rural Municipality Procedure relating to distribution of Identity Cards to People with Disabilities, 2075 | Recognition | Schedule-8 , No. 16 |  |
| 51 | Madi Rural Municipality Home Stay Operation Procedure, 2075 | Employment creation |  |  |
| 52 | Madi Rural Municipality Procedure on operation of Hotel, Home Stay, Lodge and Tea Shops in Public Places, 2076 | Employment creation |  |  |
| 53 | Madi Rural Municipality Procedure on Implementation of Integrated Model Residence Development Program, 2076 | Advance concept | Schedule-8 , No. 7 |  |
| 54 | Madi Rural Municipality Procedure on Operation of Land Plot Expansion Program, 2076 | Production development | Schedule-8 , No. 7 |  |
| 55 | Committee Formation and Operation Rule of Madi Rural Municipality, 2076 | Democratization | Article 214 |  |
| 56 | Madi Rural Municipality Gender Related Violence Control Committee Formation and Work Operation Procedure, 2076 | Equal opportunity |  |  |
| 57 | Madi Rural Municipality Fest and Festivals Operation Procedure, 2076 | Entertretment | Schedule-8 , No. 22 |  |
| 58 | Madi Rural Municipality Ambulance Service Operation Procedure, 2076 | Health Care | Schedule-8 , No. 5, 9 |  |
| 59 | Madi Rural Municipality Procedure on Formation and Management of School Management Committee, 2076 | Quality | Schedule-8 , No. 8 |  |
| 60 | Municipality Police Operation Procedure, 2076 | Security | Schedule-8 , No. 1 |  |
| 61 | Madi Rural Municipality Procedure on Operation and Management of Basic Level (Class 8) Examination | Standard meant | Schedule-8 , No. 8 |  |
| 62 | Procedure on Management of Drinking Water and Sanitation Consumer Organization, 2076 | Health Care | Schedule-8 , No. 7,21 |  |
| 63 | Madi Rural Municipality Birthplace Prize Distribution Procedure, 2076 | Motivation |  |  |
| 64 | Madi Rural Municipality Procedure relating to Brief Environmental Study and Initial Environmental Examination, 2077 | Environment portion | Schedule-8 , No. 21 |  |
| 65 | Madi Rural Municipality Procedure on Use and Management of River and Mine related Materials, 2077 | Development | Schedule-8 , No. 21 |  |
| 66 | Madi Rural Municipality Wildlife Park Operation and Management Procedure, 2077 | Ecosystem management | Schedule-8 , No. 21 |  |
| 67 | Madi Rural Municipality One Ward one Nursery Operation and Management Procedure, 2077 | Environment Conservation | Schedule-8 , No. 15 |  |
| 68 | Madi Rural Municipality Procedure relating to Selection and Recommendation of Teachers in Contract, 2077 | Qualified teacher selection | Schedule-8 , No. 8 |  |
| 69 | Madi Rural Municipality Procedure relating to operation of Agricultural Crops and Animal Husbandry Pocket Zone Program, 2077 | Conservation | Schedule-8 , No. 15 |  |
| 70 | Madi Rural Municipality Procedure Relating to Operation of Agriculture Equipment Purchase and Distribution Program, 2077 | Motivation to the farmer | Schedule-8 , No. 15 |  |
| 71 | Madi Rural Municipality Multi Year Contract Management Rule, 2078 | Quality | Schedule-8 , No. 7 |  |
| 72 | Madi Rural Municipality Internal Income Collection Contract Management Rule, 2078 | Economic development | Schedule-8 , No. 4 |  |
| 73 | Madi Rural Municipality Procedure relating to Distribution of Allowance for Fully Disabled People Caretaking, 2078 | Justice | Schedule-8 , No. 16 |  |
| 74 | Madi Rural Municipality Procedure Relating to Operation of Pregnant Women Visiting Program, 2078 | Health care | Schedule-8 , No. 8 |  |
| 75 | Madi Rural Municipality Procedure relating to Operation of Gender Related Violence Eradication Fund, 2078 | Justice |  |  |
| 76 | Madi Rural Municipality Procedure for Youth Participation in Planning Process, 2078 | Participation |  |  |
| 77 | Madi Rural Municipality Sport Development Committee Formation and Mobilization Procedure, 2078 | Health care |  |  |
| 78 | Madi Rural Municipality Mediation Center Operation Procedure, 2078 | Arbitration | Article 217 |  |
| 63 | Madi Rural Municipality Birthplace Prize Distribution Procedure, 2076 | Motivation |  |  |
| 79 | Madi Rural Municipality Procedure Related to Land Acquisition | Production |  |  |
| 80 | Working Procedure relating to functioning of Madi Rural Municipality Monitoring and Supervision Committee, 2078 | To assure consumer right and assure quality of goods and services | Article 214 |  |
| 81 | Madi Rural Municipality Youth Council Regulation Procedure, 2078 | Formation and operation of youth council |  |  |
| 82 | Madi Rural Municipality Work Operation Guidance, 2078 | To bring systematization in the functioning of local level | Article 214 |  |
| 83 | Madi Rural Municipality's Organization Registration and renew Procedure, 2078 | To register organization and renew them | Associations Registration Act, 2034, Section 4 |  |
| 84 | Madi Rural Municipality Procedure relating to Safe Immigration, 2078 | To protect the interest of local people's who are going to foreign nations |  |  |
| 85 | Procedure relating to formation and operation of Madi Rural Municipality Local Children's Club, 2078 | To empower children's and to listen their voice in public policy making | Children's Act, 2018, Section 58 |  |
| 86 | Procedure relating to Madi Rural Municipality Level President Running Shield Competition | To promote sport related activities |  |  |
| 87 | Procedure relating to Madi Rural Municipality Employee Motivation Facilities, 2078 | To motivate employees of rural municipalities for better service delivery | Article 214 |  |
| 88 | Procedure relating to Madi Rural Municipality Employee Tourism Deputation Leave, 2078 | To motivate employee through providing tourism deputation leave |  |  |

| **Table S6: Laws made by Rupa Rural Municipality during the first five years (2017-2022) of operation** | | | |
| --- | --- | --- | --- |
| **S. N** | **Name of laws** | **Objectives** | **Constitutional Local level Powers** |
| 1 | Procedure relating to ratification of decision or order of Rupa Rural Municipality, 2074 | To make publication of local level decision and laws for the knowledge of public | Article 214 |
| 2 | Code of conduct for officials of Rupa Rural Municipality, 2074 | To maintain good governance and discipline | Article 218 |
| 3 | Rupa Rural Municipalities Executive (Work Administration) Rule, 2074 | Systematize activities of executive | Article 218 |
| 4 | Procedure relating to operation of Meeting of Rupa Rural Municipality Executive, 2074 | Systematize meeting of executive | Article 218 |
| 5 | Rupa Rural Municipality Appropriation Act, 2074 | Budget formation | Article 229 |
| 6 | Rupa Rural Municipality Finance Act, 2074 | Budget formation | Article 228, 229 |
| 7 | Rupa Rural Municipality Building Construction Standard, 2074 | Helps to maintain housing quality and build long term and strong structure buildings | - |
| 8 | Rupa Rural Municipality Procedure relating to Management of Technical Staff in Contract, 2074 | Administrative management | Article214 |
| 9 | Rupa Rural Municipality Education Regulation, 2074 | To improved quality of school level education | Schedule-8 , No. 8 |
| 10 | Rupa Rural Municipality Finance Act, 2075 | Budget formation | Article 228, 229 |
| 11 | Rupa Rural Municipalities Executive (Work Division) Rule, 2075 | Systematize activities of executive | Article 218 |
| 12 | Rupa Rural Municipality Appropriation Act, 2075 | Budget formation | Article 229 |
| 13 | Rupa Rural Municipality Administrative Procedure Regulation Act, 2075 | To solve administrative problems relating to budget mobilization | Article 214 (3) |
| 14 | Rupa Rural Municipality Property Tax and Land Tax Management Act, 2075 | To broaden the revenue of local level | Schedule-8, No. 2 |
| 15 | Rupa Rural Municipality Disaster Risk Reduction and Management Act, 2075 | Institutional Preparation for disaster risk reduction and response to minimize impact | Schedule-8 , No. 20 |
| 16 | Rupa Rural Municipality Consumer Committee Formation, Mobilization and Management Procedure, 2075 | To enroll public people in development work | Schedule-8 , No. 7, 10 |
| 17 | Rupa Rural Municipality Directives for Market Monitoring , 2075 | To protect consumer rights | Schedule-8 , No. 10 |
| 18 | Rupa Rural Municipality Directives relating to distribution of Identity Cards to People with Disabilities, 2075 | Helps to provide additional facilities from local level to people with disabilities | Schedule-8 , No. 16 |
| 19 | Rupa Rural Municipality Child Right Act, 2075 | For the protection and overall development of children | Article 39 |
| 20 | Rupa Rural Municipality Directives relating to distribution of Identity Cards to Elderly Citizen , 2075 | Helps to provide additional facilities from local level to all elderly citizens living in the rural municipality | Schedule-8 , No. 16 |
| 21 | Rupa Rural Municipality Agriculture Act, 2075 | Development of agriculture Sector | Schedule-8, No. 15, 18 |
| 22 | Rupa Rural Municipality Agriculture Policy, 2075 | Development of agriculture Sector | Schedule-8, No. 15, 18 |
| 23 | Rupa Rural Municipality Directives related to maintenance Fund Operation, 2075 |  |  |
| 24 | 'D" Class Construction Business License Procedure 2075 | Registration and monitoring construction work |  |
| 25 | Rupa Rural Municipality Health and Sanitation Act, 2075 | To protect public health and maintain surrounding clean and hygienic | Schedule-8, No. 9 |
| 26 | Rupa Rural Municipality Procedure relating to organization of Program on awareness against Child Marriage, 2075 | To stop and minimize Child Marriage | Article 39 |
| 27 | Rupa Rural Municipality Finance Act, 2076 | Budget formation | Article 228, 229 |
| 28 | Rupa Rural Municipality Appropriation Act, 2076 | Budget formation | Article 228, 229 |
| 29 | Rupa Rural Municipality Procedure on One Village one Agricultural Production Program | To increase agricultural production | Schedule-8, No. 15, 18 |
| 30 | Rupa Rural Municipality Procedure on operation of Program on distribution of grant for Production and delivery of Milk, Vegetable and Fruits , 2076 | To increase agricultural production | Schedule-8, No. 15, 18 |
| 31 | Rupa Rural Municipality Procedure on Operation of Land Plot Expansion Program, 2076 | To use land on productive activities | Schedule-8 , No. 7 |
| 32 | Rupa Rural Municipality Procedure on Operation of Program on Bartering of Goats, 2076 | To help the poor farmers | Schedule-8 , No. 15 |
| 33 | Rupa Rural Municipality Standard on Use and Management of Covid isolation Fund | To control spread of Covid-19 | Schedule-8, No. 9 |
| 34 | Rupa Rural Municipality Procedure on Improvement of Animals Breed, 2077 | Increase in productivity of Animals | Schedule-8 , No. 15 |
| 35 | Rupa Rural Municipality Procedure relating to distribution of grant to small scale farmers , 2077 | Development of agriculture Sector , increase in agricultural yield and income of farmers | Schedule-8 , No. 15, 18 |
| 36 | Rupa Rural Municipality Finance Act, 2078 | Budget formation | Article 228, 229 |
| 37 | Rupa Rural Municipality Appropriation Act, 2078 | Budget formation | Article 228, 229 |
| 38 | Rupa Rural Municipality Cooperative Act, 2077 | Regulation of Cooperatives | Schedule-8, No. 2 |
| 39 | Rupa Rural Municipality Organization registration and Renew Act, 2077 | Registration and renew of organizations | Associations Registration Act, 2034, Section 4 |
| 40 | Rupa Rural Municipality Standard for making expenditure, 2077 | Promote good governance and economic transparency | Article 229 |
| 41 | Rupa Rural Municipality Rule on Public Procurement | Promote good governance and economic transparency | Article 229 |
| 42 | Rupa Rural Municipality Procedure on Monitoring of Plan and Program implementation and systematizing the Payment Process, 2077 | Promote good governance and economic transparency | Article 229 |
| 43 | Rupa Rural Municipality Procedure on Operation and Management of Agricultural Enterprise, 2077 | Development of agriculture Sector , increase in agricultural yield and income of farmers | Schedule-8 , No. 15, 18 |
| 44 | Rupa Rural Municipality Procedure on Operation of program on Land Bank, 2077 | Wise utilization of land resources | Schedule-8 , No. 5, 15, 18 |
| 45 | Directives relating to Operation of program relating to Animal Husbandry, 2077 | Promoting animal health and increasing productivity from animals | Schedule-8 , No. 15 |
| 46 | Rupa Rural Municipality Procedure relating to joint program with teachers on one teacher one laptop handover program for promoting technology in education, 2077 | Improving quality of education and motivating teachers | Schedule-8 , No. 8 |
| 47 | Rupa Rural Municipality Regulation relating to implementation of program related with agriculture development program, 2077 | Development of agriculture Sector , increase in agricultural yield and income of farmers | Schedule-8 , No. 15, 18 |
| 48 | Rupa Rural Municipality Cooperative Act, 2077 | Regulation of Cooperatives | Schedule-8, No. 2 |
| 49 | Rupa Rural Municipality Procedure on Operation of program on Social Inclusiveness, 2078 | Promoting inclusiveness | Article 42, 43 |
| 50 | Rupa Rural Municipality Procedure on Operation of Revenue Advisory Committee, 2078 | To increase tax base for making fund available for development activities | Article 229, Schedule-8, No. 7 |
| 51 | Rupa Rural Municipality Procedure relating to operation of functioning of committee on Budget drafting and program drafting committee, 2078 | Making plan and programs effective for overall development of municipality | Article 229, Schedule-8, No. 7 |
| 52 | Rupa Rural Municipality Procedure relating to Gender Equality and Inclusiveness, 2078 | Promoting gender equality and inclusiveness | Article 42, 43 |
| 53 | Rupa Rural Municipality Finance Act, 2078 | Budget formation | Article 228, 229 |
| 54 | Rupa Rural Municipality Appropriation Act, 2078 | Budget formation | Article 228, 229 |
